# Optimising outcomes in lateral unicompartmental knee arthroplasty: Analysing 25 years of registry data

**DOI:** 10.1002/ksa.12785

**Published:** 2025-07-13

**Authors:** Kristine Ifigenia Bunyoz, Martin Lindberg‐Larsen, Kirill Gromov, Anders Troelsen

**Affiliations:** ^1^ Department of Orthopaedic Surgery Copenhagen University Hospital Hvidovre Hvidovre Denmark; ^2^ Department of Health and Medical Science Copenhagen University Copenhagen Denmark; ^3^ Department of Clinical Research University of Southern Denmark Odense Denmark; ^4^ Department of Orthopaedic Surgery and Traumatology Odense University Hospital Odense Denmark; ^5^ Department of Clinical Medicine Copenhagen University Copenhagen Denmark

**Keywords:** lateral UKA, outcome analysis, unicompartmental knee arthroplasty, registry data, survival

## Abstract

**Purpose:**

Limited data and experience surround lateral unicompartmental knee arthroplasty (UKA), contributing to uncertainty regarding its broader application in clinical practice. To understand how to optimise lateral UKA outcomes, this study aimed to evaluate the implant survival of lateral UKA and compare it to total knee arthroplasty (TKA) using registry data.

**Methods:**

Data were obtained from the Danish Knee Arthroplasty Registry, the Danish National Patient Registry and the Danish Civil Registration System. Between 1997 and 2022, all primary lateral UKAs (*n* = 538) and primary TKAs performed on valgus‐aligned knees were included. Propensity score matching (1:4) matched patients by age, sex, weight, Charlson comorbidity index, year of surgery and hospital type. Survival analysis used the Fine‐Gray subdistribution hazards model to account for competing risks.

**Results:**

The 5‐year cumulative revision risk was 10.1% for lateral UKA and 5.0% for TKA (1997–2022). For lateral UKA, this decreased from 25.0% (1997–2006) to 7.3% (2017–2022); TKA decreased from 4.6% to 3.7%. Surgery after 2011 and use of the fixed lateral Oxford (FLO) implant significantly reduced the risk of revision. Compared to TKA, the subdistribution hazard ratio for revision was 0.7 (95% confidence interval [CI] = 0.2–2.2) for the FLO implant and 3.4 (95% CI = 1.9–6.1) for other lateral UKAs in the period 2017–2022. No differences were found in 90‐day readmissions or complications between lateral UKA and TKA, but the 2‐year reoperation rate was significantly lower for lateral UKA in both periods.

**Conclusion:**

Lateral UKA survival has evolved with improved understanding of knee compartment biomechanics, indications, surgical techniques and implant designs. Lateral UKA with the FLO implant showed lower or similar revision rates compared with TKA.

**Level of Evidence:**

Level III.

**Abbreviations**
CCI: Charlson comorbidity index
CI: confidence interval
FLO: fixed lateral Oxford
ICD: International Statistical Classification of Diseases and Related Health Problems diagnosis codes
OA: osteoarthritis
PFA: patellofemoral arthroplasty
sHR: subdistribution hazard ratio
SMD: standardised mean difference
TKA: total knee arthroplasty
UKA: unicompartmental knee arthroplasty
VIF: variance inflation factor

## INTRODUCTION

Unicompartmental knee arthroplasty (UKA) has gained popularity in recent years due to advancements in implant design and surgical techniques, offering several benefits over total knee arthroplasty (TKA) [6, 9, 14, 20, 22]. These benefits include preserving more of the native joint, allowing for more natural knee kinematics, faster recovery and reduced morbidity and mortality, making UKA an appealing option for patients with isolated compartmental osteoarthritis (OA) [6, 9, 14, 20, 22]. Despite the widespread adoption of medial UKA, lateral UKA remains potentially underutilized [2, 7, 23], even though it appears to offer similar potential for treating lateral compartment OA when used in well‐established high‐usage UKA centres [1, 13, 29, 30].

The less common practice of lateral UKA may be explained by several factors: the lack of large‐scale multicentre studies evaluating its outcomes, the perceived complexity of the surgical technique and the absence of consensus and evidence‐based indications for lateral UKA [10]. These factors may discourage some surgeons from adopting lateral UKA into routine practice. Additionally, lateral compartment OA being less common than medial compartment OA may reduce surgeons' opportunity to develop expertise in lateral UKA.

Most research on lateral UKA consists of smaller, single‐centre studies, which limits the ability to draw robust, generalisable conclusions about outcomes. However, the studies using fixed‐bearing implants report excellent patient satisfaction and high survival rates after lateral UKA [1, 3, 13, 29], suggesting that lateral UKA can be a highly effective treatment option with proper patient selection and a fixed‐bearing implant.

Nonetheless, the limited data and experience surrounding lateral UKA create uncertainty about its broader application in clinical practice. To bridge this gap and provide more definitive insights, we conducted a registry‐based study to deliver more comprehensive data on the performance and outcomes of lateral UKA on a national scale.

The overall aim was to evaluate the survival of lateral UKA from its initial registration in the Danish Knee Arthroplasty registry in 1997 through to the end of 2022, using TKA as the comparative procedure, given its role as the alternative surgical procedure for treating end‐stage lateral OA.

The specific study objectives were:

1. To compare the cumulative risk and reasons for revision for TKA and lateral UKA over the entire study period (1997–2022).
2. To evaluate changes in the cumulative risk of revision over time from 1997 to 2022.
3. To compare 90‐day readmissions, 90‐day complications and 2‐year reoperations between TKA and lateral UKA.
4. Identify factors contributing to the optimal survival of lateral UKA.

We hypothesise that the cumulative risk of revision is higher for lateral UKA than for TKA over the entire study period, but that lateral UKA survival has improved over time with advancements in implant design and increased utilisation.

## MATERIALS AND METHODS

This study adheres to the Strengthening the Reporting of Observational Studies in Epidemiology guidelines and incorporates the Reporting of studies Conducted using Observational Routinely‐collected Data guidelines.

### Data process

Data were obtained from the Danish Knee Arthroplasty Registry, including all primary and revision TKAs, medial UKAs and lateral UKAs from 1997 to 2022. These were linked to the Danish National Patient Registry (comorbidities, readmissions and reoperations) and the Danish Civil Registration System (mortality and emigration status).

We included all primary lateral UKAs and only primary TKAs performed on valgus‐aligned knees (4–8 degrees valgus) to ensure a fair comparison between groups.

**Figure 1:**
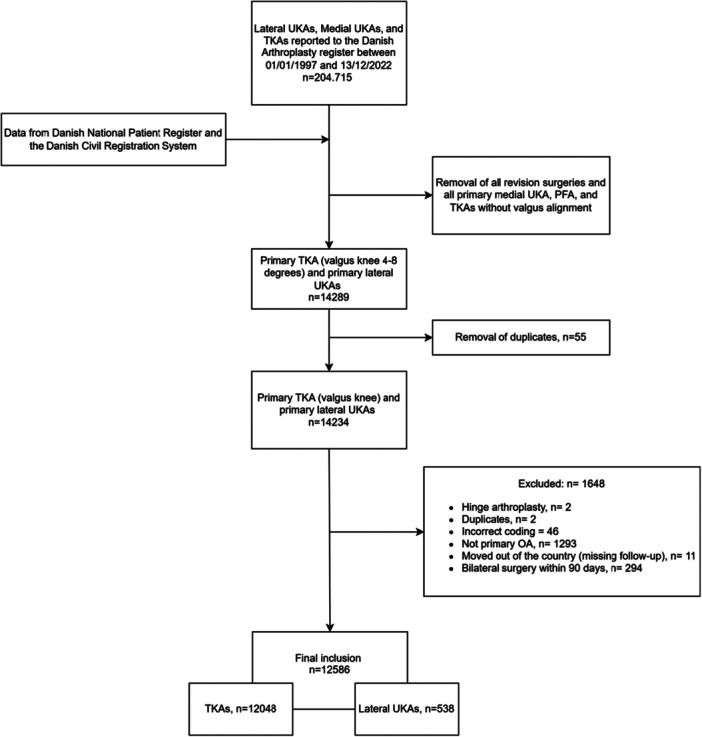
Flowchart of patient selection. OA, osteoarthritis; PFA, patellofemoral arthroplasty; TKA, total knee arthroplasty; UKA, unicompartmental knee arthroplasty.

Exclusions included surgeries for fractures, arthritis, cancer, haemophilia or unknown indications; patients lost to follow‐up due to emigration; and those with bilateral knee arthroplasties within 90 days. This yielded 12,586 procedures (12,048 TKAs and 538 lateral UKAs) for analysis. See Figure 1 for a detailed flowchart.

### Outcome measures and definitions

Charlson comorbidity index (CCI) was calculated using the International Statistical Classification of Diseases and Related Health Problems (ICD‐8 and ICD‐10) diagnosis from the Danish National Patient Registry, based on hospital contacts within 10 years prior to surgery.

Ninety‐day *readmissions* were defined as hospital admissions lasting ≥1 day, recorded from the day following surgery. Post‐operative *complications* within 90 days were identified using predefined ICD‐10 codes [11] (Appendix S1, Table 1).

*Two‐year reoperations* were identified through predefined ICD‐10 procedural codes categorised as: manipulation under anaesthesia, infection treated with debridement, the addition of a medial UKA, bleeding, wound rupture, fracture and unspecific (Appendix S1, Table 2).

*Revisions* were identified through the Danish Knee Arthroplasty Registry. To capture all additions of medial UKAs following lateral UKAs, these were searched as both revisions and primary surgeries. In analyses, such additions were treated as reoperations, not revisions.

*Usage* was defined as the percentage of UKAs among all primary knee arthroplasties, calculated per hospital per calendar year. High usage was defined as >20%.

### Subanalysis with the fixed lateral Oxford (FLO) implant

The FLO implant (Zimmer Biomet) was first registered in the Danish Knee Arthroplasty Registry in 2017. Since then, it has become the predominant fixed‐bearing design for lateral UKA in Denmark, accounting for 54.4% (*n* = 190) of all lateral UKAs implanted. Given its prevalence, the FLO implant was included as an individual covariate in the analysis of factors influencing lateral UKA survival.

### Statistical analysis

Propensity score matching at a 1:4 ratio was used to match lateral UKA and TKA patients by age, sex, weight, CCI, year of surgery and hospital type. Missing data for weight (*n* = 283) and CCI (*n* = 4) were addressed using multiple imputation under the assumption of missing at random. Balance between groups was assessed using a standardised mean difference (SMD), with ≤0.1 indicating adequate balance.

Descriptive statistics were used to summarise patient characteristics. Logistic regression compared 90‐day readmissions, 90‐day complications and 2‐year reoperations. Adjustment for covariates (age, sex, CCI, year of surgery and hospital type) was applied to 90‐day readmissions; adjusted models were not performed for complications or reoperations due to low event counts. Model fit was evaluated with residual plots, and multicollinearity was assessed using variance inflation factors (VIFs), with a VIF > 5 indicating collinearity.

Survival analysis was performed using the Fine‐Gray subdistribution hazards model to account for death as a competing risk. Covariates included age, sex, CCI, year of surgery, UKA usage and implant type (FLO vs. other lateral UKAs). Subdistribution hazard ratios (sHRs) with 95% confidence intervals (CIs) were reported, and cumulative incidence functions were used to illustrate revision risk over time. Statistical significance was set at *p* < 0.05.

All analyses were performed in RStudio (version 2024.04.2) using the mice, MatchIt, cmprsk and mets packages.

## RESULTS

### Patient demographics

All matching variables were adequately balanced. The mean age was approximately 67 years, over 70% were female, over 60% had a CCI of 0, and over 90% of surgeries were performed in public hospitals (Table 1).

**Table 1:** Patient demographics—Comparison of crude and propensity‐scored matched data.

	Crude data			1:4 matched data		
	LAT UKA, *n* = 538	TKA (valgus), *n* = 12,048	SMD	LAT UKA, *n* = 538	TKA (valgus), *n* = 2152	SMD
Age at surgery, mean years (range)	66.7 (28–90)	70.2 (26–95)	0.312	66.7 (28–90)	67.4 (26–92)	0.063
Sex (male), *n* (%)	158 (29.4)	2788 (23.1)	0.142	158 (29.4)	577 (26.8)	0.057
Weight, mean kg (range)	82.3 (48–176)	82.6 (40–200)	0.017	82.3 (48–176)	81.7 (45–186)	0.031
Charlson comorbidity index, *n* (%)			0.090			0.039
0: None	362 (67.3)	7609 (63.2)		362 (67.3)	1411 (65.6)	
1–2: Mild	139 (25.8)	3547 (29.4)		139 (25.8)	589 (27.4)	
3–4: Moderate	29 (5.4)	726 (6.0)		29 (5.4)	116 (5.4)	
≥5: Severe	8 (1.5)	166 (1.4)		8 (1.5)	36 (1.7)	
Year of surgery, *n* (%)			0.914			0.053
1997–2006	8 (1.5)	2789 (23.1)		8 (1.5)	22 (1.0)	
2007–2011	74 (13.8)	2931 (24.3)		74 (13.8)	276 (12.8)	
2012–2016	107 (19.9)	2665 (22.1)		107 (19.9)	423 (19.7)	
2017–2022	349 (64.9)	3663 (30.4)		349 (64.9)	1431 (66.5)	
Unit type (public), *n* (%)	506 (94.1)	10,848 (90.0)	0.149	506 (94.1)	2011 (93.4)	0.025
*Non‐matched variables*						
Side (right), *n* (%)	337 (62.6)	7122 (59.0)		337 (62.6)	1205 (56.0)	
AKSS function, mean (SD)	43.4 (25.6)	47.9 (19.3)		43.4 (25.6)	48.9 (18.7)	
Fixation, *n* (%)						
Cemented	312 (58.0)	8981 (74.5)		312 (58.0)	1446 (67.2)	
Cementless	8 (1.5)	1028 (8.5)		8 (1.5)	270 (12.5)	
Hybrid	215 (40.0)	2005 (16.6)		215 (40.0)	430 (20.0)	
Missing	3 (0.6)	34 (0.3)		3 (0.6)	6 (0.3)	
Duration of surgery, mean min (range)	71.9 (32–170)	69.2 (25–270)		71.9 (32–170)	66.0 (31–180)	

Abbreviations: AKSS, American Knee Society Score (function subscale); LAT UKA, lateral unicompartmental knee arthroplasty; SD, standard deviation; SMD, standard mean difference; TKA, total knee arthroplasty.

### Cumulative risk and reasons for revision for TKA and lateral UKA

**Figure 2:**
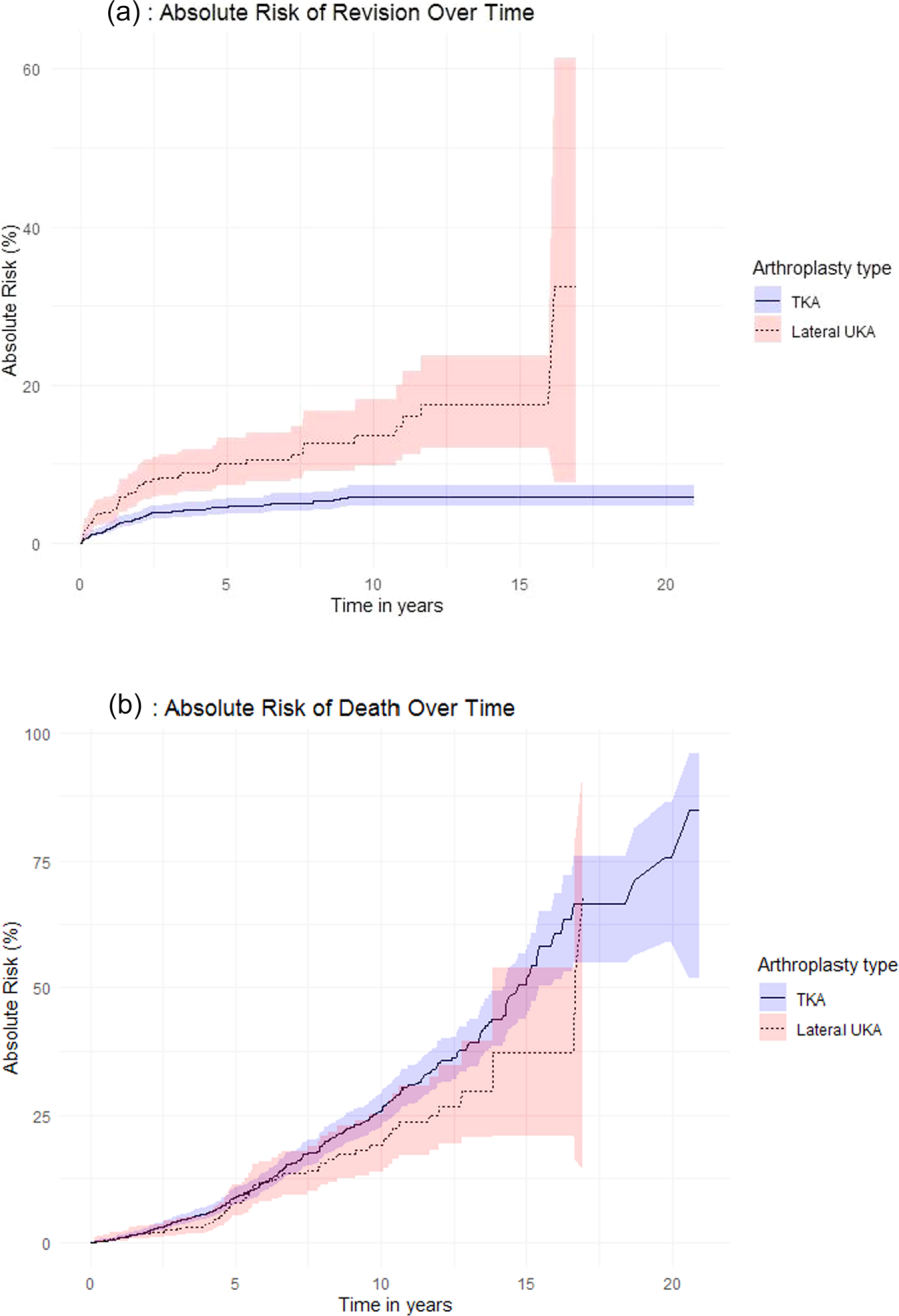
(a, b) Absolute cumulative risk (%) of revision and death for lateral UKA and TKA (1997–2022). The absolute cumulative risk of revision and death for all primary lateral UKAs and TKAs performed for primary osteoarthritis between 1997 and 2022, illustrated with 95% CI. CI, confidence interval; TKA, total knee arthroplasty; UKA, unicompartmental knee arthroplasty.

With death as a competing risk, the 10‐year cumulative risk of revision for lateral UKA and TKA over the study period (1997–2022) was 13.6% and 5.9%, respectively (Figure 2a,b, Appendix S2, Table 1).

The unadjusted sHR for lateral UKA was 2.5 (95% CI = 1.8–3.5) for revision and 0.7 (95% CI = 0.6–0.99) for death. After adjusting for age, sex, CCI and year of surgery, the sHR for revision decreased to 2.3 (95% CI = 1.6–3.2), and the sHR for death increased to 0.8 (95% CI = 0.6–1.1).

During the full study period, 52 knees (9.7%) with lateral UKA and 92 knees (4.3%) with TKA underwent revision surgery. In the lateral UKA group, the primary reasons for revision were aseptic loosening, pain without loosening, infection, and progression of OA. For the TKA group, instability and infection were the most common causes. A comprehensive list of revision reasons with statistics is available in Table 2.

**Table 2:** Reasons for revision (1997–2022).

Reason for revision, *n* (%)	Lateral UKA (*n* = 538), *n* (%)	TKA (*n* = 2152), *n* (%)
Aseptic loosening	6 (1.1)	10 (0.5)
Pain without loosening	12 (2.2)	15 (0.7)
Instability	5 (0.9)	29 (1.4)
Infection	7 (1.3)	21 (1.0)
Add on of patella component	0 (0.0)	2 (0.1)
Polyethylene failure	3 (0.6)	0 (0.0)
Progression of osteoarthritis	8 (1.5)	1 (0.1)^a^
Fracture	0 (0.0)	4 (0.2)
Tendon rupture	1 (0.2)	0 (0.0)
Decreased ROM	0 (0.0)	4 (0.2)
Luxation of bearing	5 (0.9)	0 (0.0)
Capsular rupture	0 (0.0)	1 (0.1)
NA	5 (0.9)	5 (0.2)

*Note*: In addition to the above revisions, one patient experienced an add‐on of a medial UKA within the study period (>2 years from the primary surgery) due to progression of osteoarthritis.

Abbreviations: ROM, range of motion; TKA, total knee arthroplasty; UKA, unicompartmental knee arthroplasty.

^a^ Resurfacing of the patella.

### Changes in the cumulative risk of revision over time

**Figure 3:**
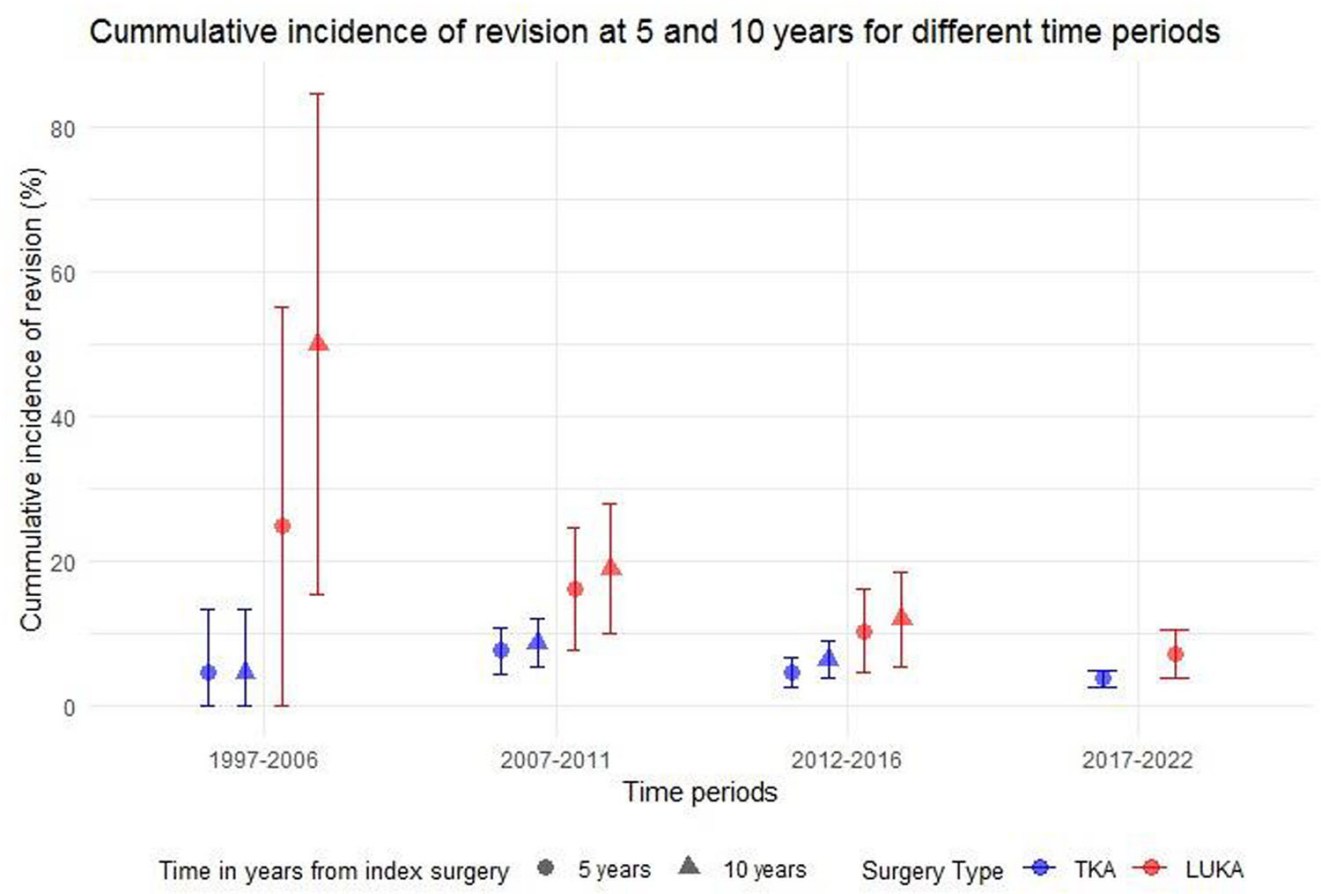
Cumulative incidence of revision for different time periods. Cumulative incidence of revision at 5 and 10 years with 95% confidence intervals (CIs) for primary total knee arthroplasty (TKA) and lateral unicompartmental knee arthroplasty (LUKA) for the four time periods (1997–2006, 2007–2011, 2012–2016, 2017–2022).

The 5‐year cumulative risk of revision for lateral UKA declined from 25.0% (1997–2006) to 7.3% (2017–2022), while TKA experienced a smaller reduction from 4.6% to 3.7% (Figure 3).

Despite the improvement in lateral UKA survival over the years, the risk of revision remained significantly higher for lateral UKA than TKA across all periods (Appendix S2, Table 2).

### 90‐day readmissions, 90‐day complications and 2‐year reoperations

The unadjusted and adjusted odds ratios (ORs) for 90‐day readmissions and the unadjusted OR for 90‐day complications showed no significant differences between the groups (Table 3). These findings remained consistent in surgeries performed from 2017 to 2022.

**Table 3:** Ninety‐day readmissions, 90‐day complications and 2‐year reoperations.

	TKA		Lateral UKA			
	*n*	% (CI)	*n*	% (CI)	Unadjusted OR (CI)	Adjusted OR (CI)
1997–2022						
90‐day readmissions	169	7.9 (6.8–9.1)	48	8.9 (6.7–11.7)	1.1 (0.8–1.6)	1.2 (0.8–1.6)^a^
90‐day complications	172	8.0 (6.9–9.2)	44	8.2 (6.0–10.8)	1.0 (0.7–1.4)	
2‐year reoperations	90	4.2 (3.4–5.1)	6	1.1 (0.5–2.4)	**0.3 (0.1–0.6)**	
2017–2022						
90‐day readmissions	101	7.05 (5.79–8.51)	29	8.31 (5.65–11.71)	1.2 (0.8–1.8)	1.2 (0.7–1.8)^b^
90‐day complications	105	7.34 (6.04–8.81)	26	7.4 (4.92–10.72)	1.0 (0.6–1.6)	
2‐year reoperations	49	3.42 (2.60–4.50)	2	0.57 (0.16–2.07)	**0.2 (0.03–0.5)**	

*Note*: ORs with 95% CI based on logistic regressions comparing lateral UKA and TKA patients. Bold values indicate OR with 95% CI not including 1 (statistically significant).

Abbreviations: CI, confidence interval; OR, odds ratio; TKA, total knee arthroplasty; UKA, unicompartmental knee arthroplasty.

^a^ Adjusted for age, sex, Charlson comorbidity index, time of surgery and unit type.

^b^ Adjusted for age, sex, Charlson comorbidity index and unit type.

Pulmonary embolism and deep vein thrombosis were the most common medical complications, occurring in 1.1% of TKA and 1.7% of lateral UKA cases. The most frequent early surgical complications were mechanical issues (3.1% for TKA, 2.0% for lateral UKA) and infections (1.8% for TKA, 2.0% for lateral UKA) (Appendix S2, Table 3).

Reoperations within 2 years occurred in 4.2% of TKA and 1.1% of lateral UKA cases. Lateral UKA had significantly lower 2‐year reoperation rates over both the full study period (OR = 0.3, 95% CI = 0.1–0.6) and the most recent period (OR = 0.2, 95% CI = 0.0–0.6) (Table 2; Appendix S2, Table 4).

### Factors contributing to optimal survival of lateral UKA

Surgery after 2011 (2012–2016: *p* = 0.011; 2017–2022: *p* = 0.031) and use of the FLO implant (*p* = 0.011) were linked to a lower revision risk after lateral UKA, while a CCI of 3–4 increased the risk (*p* = 0.048) (Table 4).

**Table 4:** The effect of covariates on the hazard ratios of experiencing revision after lateral UKA.

Risk factors	sHR	*p*
Age <70 years	Reference	
Age ≥70 years	0.7 (0.4–1.3)	0.207
Female sex	Reference	
Male sex	1.1 (0.6–1.9)	0.863
CCI (none)	Reference	
CCI (mild)	1.3 (0.6–2.5)	0.515
CCI (moderate)	2.8 (1.0–7.6)	**0.048**
CCI (severe)	1.7 (0.4–8.1)	0.519
Operation date 1997–2006	Reference	
Operation date 2007–2011	0.4 (0.2–1.2)	0.102
Operation date 2012–2016	0.3 (0.1–0.7)	**0.011**
Operation date 2017–2022	0.3 (0.1–0.9)	**0.031**
UKA usage <20%	Reference	
UKA usage >20%	1.1 (0.5–2.3)	0.789
Lateral UKA using any other implant than FLO	Reference	
Lateral UKA using FLO	0.20 (0.1–0.7)	**0.011**

*Note*: sHR calculated using the Fine‐Grey competing risk methods. Bold values indicate statistically significant (*p* < 0.05)

Abbreviations: CCI, Charlson comorbidity index; FLO, fixed Oxford lateral implant; sHR, subdistribution hazard ratio; UKA, unicompartmental knee arthroplasty.

**Figure 4:**
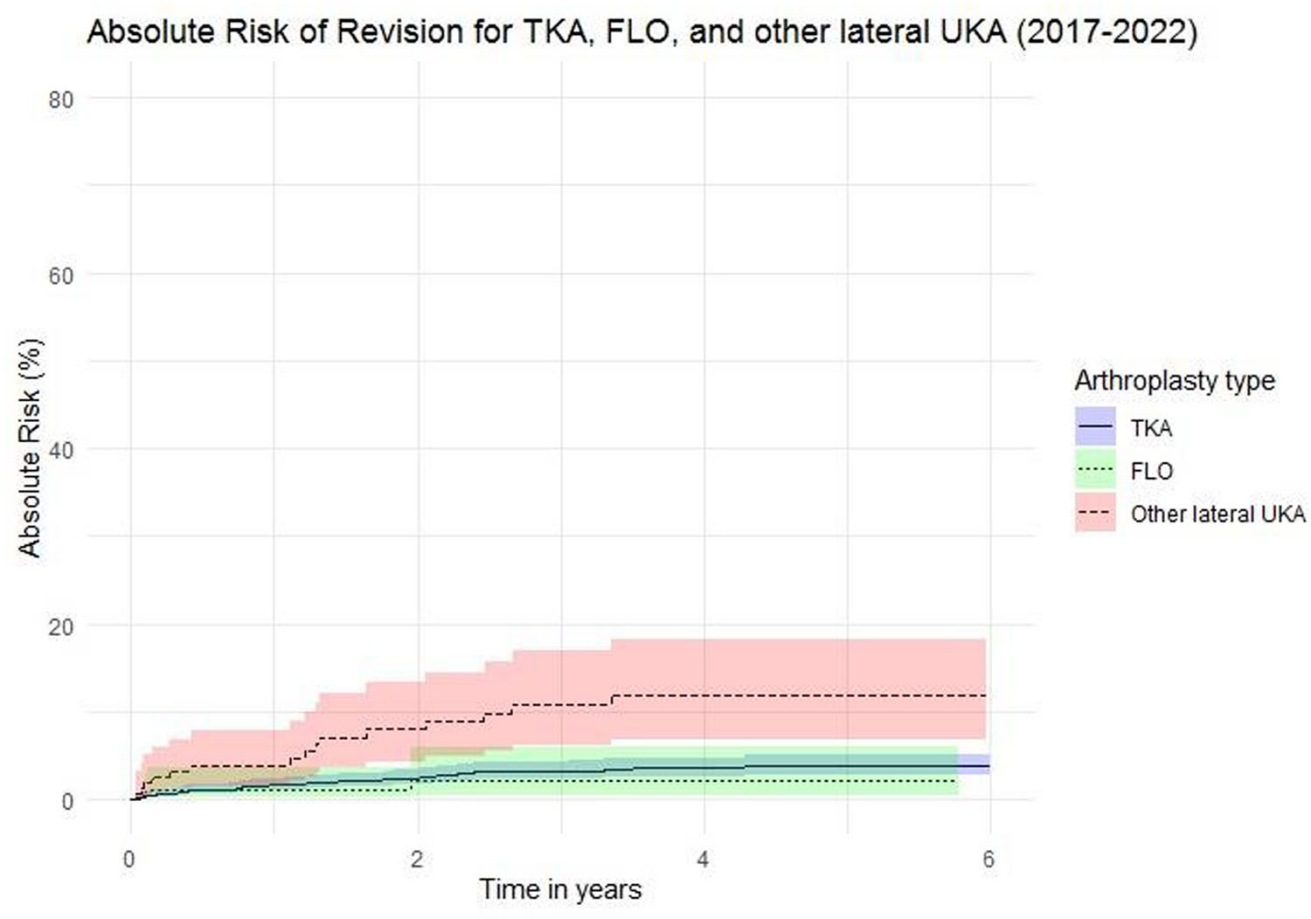
Absolute risk of revision for total knee arthroplasty (TKA), fixed lateral Oxford (FLO) and other lateral unicompartmental knee arthroplasty (UKA) from 2017 to 2022. The survival curve shows the absolute risk of revision for TKA, the FLO and all other lateral UKAs from 2017 to 2022. No significant differences were found between the risk of revision after TKA and FLO (*p* = 0.508). A significantly higher risk of revision was found between TKA and other lateral UKA implants (*p* = 0.0001).

In 2017–2022, using TKA (*n* = 1409) as reference, the sHR for revision was 0.7 (95% CI = 0.2–2.1, *p* = 0.508) for FLO implants (*n* = 190) and 3.4 (95% CI = 1.9–6.1, *p* = 0.0001) for other lateral UKAs (*n* = 159). Adjusting for age, sex and CCI did not change these estimates (Figure 4).

## DISCUSSION

This study found that: (1) The absolute cumulative risk of revision was significantly higher for lateral UKA compared to TKA over the entire study period (1997–2022). However, lateral UKA survival improved over time, particularly for procedures performed after 2011. (2) No significant differences were observed in 90‐day readmission or complication rates between TKA and lateral UKA, but the 2‐year reoperation rate was significantly lower for lateral UKA. (3) The FLO implant and surgeries performed after 2011 were significantly associated with a lower risk of revision after lateral UKA. No significant difference in risk of revision was observed between the FLO implant and TKA.

The outcomes and survival of lateral UKA have been investigated more intensively in cohort studies, often reporting excellent results, especially when performed in high UKA usage centres using fixed‐bearing implants [1, 3, 13, 19, 26, 27, 29]. However, to our knowledge, only three prior studies [2, 4, 17] have used registry data to investigate lateral UKA outcomes, and historically, registries have not distinguished between medial and lateral UKAs in their annual reports. Mohammad et al. [17] reported 88.6% 10‐year survival for Oxford domed lateral UKAs (2005–2017) without comparisons. Mobile‐bearing lateral UKAs have historically been associated with higher revision rates, possibly negatively affecting survival [5, 24]. Baker et al. [2] found 93.0% 7‐year survival (2003–2010), with older age reducing revision risk; outcomes matched medial UKA. Both studies [2, 17] used National Joint Registry data and Kaplan–Meier analysis, which does not account for death as a competing risk, potentially overestimating revision rates. Burger et al. [4] incorporated competing risk analysis, reporting a 5‐year revision rate of 12.6% (2007–2017), with higher risk for mobile‐bearing implants; results were also compared to medial UKA.

Our study found a 13.6% 10‐year cumulative revision risk for lateral UKA (1997–2022), with the 5‐year risk decreasing from 25% before 2007 to 7.3% after 2017, likely reflecting advances in surgical technique, implant design and biomechanics. Furthermore, lateral UKAs are often done in high‐volume centres experienced in medial UKA, enhancing outcomes. The FLO implant demonstrated a notably low 5‐year cumulative revision risk of 2.1%, possibly due to its use by surgeons highly familiar with medial UKA and the reamer‐based femoral preparation allowing precise lateral compartment reconstruction in extension.

Unlike the previous registry studies [2, 4], we compared lateral UKA to TKA—the primary treatment options for end‐stage lateral OA. Lateral UKA showed a higher revision risk than TKA throughout the study period, though this gap narrowed over time with advancements in surgical technique and implant design. Although not statistically significant after adjustment, the cumulative risk of death was lower for lateral, aligning with existing literature, suggesting lower mortality after UKA compared to TKA [9, 14, 20]. The consistently higher revision risk following lateral UKA may also reflect a lower threshold for revision, given the relative ease of revising UKA and the generally better outcomes of these revisions (revision bias) [12, 18, 21, 25].

While 90‐day readmissions and complications did not differ significantly between TKA and lateral UKA, the 2‐year reoperation rate was significantly lower for lateral UKA (OR = 0.3, 95% CI = 0.1–0.6), supporting its broader use in treating end‐stage lateral OA. Most existing studies comparing lateral UKA and TKA are short term [8, 15, 26, 28], typically showing faster recovery and better short‐term functional outcomes after lateral UKA, while TKA tends to have lower revision rates, though these differences are not significant. Only one randomised controlled trial (*n* = 54) [8] has compared lateral UKA and TKA. This recent Chinese study found significantly lower operating time, post‐operative haemoglobin reduction, pain scores, hospital stay, and a higher 1‐year Forgotten Joint Scores with lateral UKA, reinforcing the benefits of UKA [6, 9, 14, 20, 22].

Our study has several limitations. Registry data may be affected by inaccurate or incomplete entries, potentially influencing findings. However, the Danish Knee Arthroplasty Registry is a nationwide database with mandatory reporting and high completeness (92.8%–97.6%, since 2016), minimising missing data.

The non‐randomised design introduces potential selection bias and unmeasured confounding. Surgical indications may vary with surgeon experience, and more experienced surgeons may apply broader UKA indications. Lack of data on surgeon experience, alignment correction, implant positioning and rehabilitation is a limitation, as these may influence outcomes. As with any long‐term registry study, shorter follow‐up in recent years may underestimate revision rates. This, however, applies equally to both groups, and the year of surgery was included in the PSM to ensure comparable follow‐up.

Complications and reoperations were identified using predefined diagnosis and procedure codes, which may not be exhaustive. The sHR for revision of the FLO implant versus TKA from 2017 to 2022 was 0.7 (95% CI = 0.2–2.1). As the tibial component is monoblock, it is not replaced during reoperation for acute infection, which may contribute to a lower reported revision rate. However, these cases should be captured as early reoperations. Finally, the lack of detailed analysis by implant type is a limitation, as design differences may impact survivorship and clinical outcomes.

Despite its limitations, the study's large cohort strengthens its ability to assess rare outcomes like revisions and reoperations, enhances generalisability, and includes patients from both private and public sectors. Furthermore, the multi‐decade timeframe enables the reflection of improvement in surgical techniques and implant design. We believe these advantages outweigh the limitations associated with registry‐based research. To ensure a valid comparison between TKA and lateral UKA, the TKA cohort was restricted to patients with valgus alignment similar to typical lateral UKA candidates, likely excluding severe valgus or fixed deformities. This is a key strength, as OA patterns and alignment differences are often overlooked in registry studies. Propensity score matching further reduced baseline imbalances, supporting the validity of our findings.

We believe the findings of this study provide a valuable addition to already published literature on lateral UKA with insights into optimising outcomes and the evolving role of lateral UKA in the management of end‐stage lateral OA. The improved survival rates after 2011 and the low revision risk with the FLO implant highlight the benefits of modern surgical techniques, implant design, and biomechanical understanding. These findings imply that with careful patient selection and the use of contemporary implants, lateral UKA can offer a viable alternative to TKA. Particularly for younger or active patients, it may also delay the need for further treatment, such as TKA. Furthermore, some of the observed differences in revision thresholds may reflect a need to re‐evaluate clinical decision‐making around the timing and indications for revision surgery, which is potentially biased in UKA. Additionally, this study implies that conclusions about lateral UKA based on registry data should exclude surgeries performed before 2012, as they reflect historical rather than contemporary outcomes. The importance of focusing on data from modern practice with appropriate UKA use has previously been highlighted [16].

## CONCLUSIONS

In conclusion, lateral UKA has evolved with an improved understanding of knee compartment biomechanics, indications, surgical techniques, and implant designs. This study demonstrated that lateral UKA performed after 2017 using the FLO implant represents a feasible treatment option for end‐stage lateral OA, as the FLO implant showed lower or similar revision rates compared with TKA. Contemporary outcomes and implants coupled with the established benefits of UKA establish lateral UKA as a feasible treatment option for eligible patients.

## AUTHOR CONTRIBUTIONS

**Kristine Ifigenia Bunyoz**: Conceptualisation; formal analysis; data curation; visualisation; writing—original draft. **Martin Lindberg‐Larsen**: Conceptualisation; writing—review and editing; supervision. **Kirill Gromov**: Conceptualisation; writing—review and editing; supervision. **Anders Troelsen**: Conceptualisation; writing—review and editing; supervision.

## CONFLICTS OF INTEREST STATEMENT

Anders Troelsen and Kirill Gromov have received research support from Zimmer Biomet, and Anders Troelsen from Pfizer Denmark. Anders Troelsen is a consultant to Zimmer Biomet and Pfizer Denmark and has received speaker honoraria. Anders Troelsen is a board member of *The European Knee Society*. Martin Lindberg‐Larsen is chairman of the steering committee of the Danish Knee Arthroplasty Register. Kristine Ifigenia Bunyoz has received travel support from ZimmerBiomet to attend the Oxford Knee Course and from Clockwork Medical to attend the Oxford Bristol Knee Meeting.

## ETHICS STATEMENT

The project was registered and approved by The Knowledge Centre on Data Protection Compliance in the Capital Region of Denmark (Privacy approval number: P‐2022‐837). No approval from regional or national research ethical committees was required for this study.

## Supporting information

Appendix S1.

Appendix S2.

## Data Availability

After application, the data supporting this study's findings were obtained from the Danish Knee Arthroplasty Registry, the Danish National Patient Registry and the Danish Civil Registration System. Access is restricted, and a license was granted for this study. Therefore, the data cannot be shared but may be requested directly from these institutions.
