# Supplementary material for: Optimising outcomes in lateral unicompartmental knee arthroplasty: Analysing 25 years of registry data
Source: Knee Surg Sports Traumatol Arthrosc. 2025 Jul 13;33(12):4324–34. doi: 10.1002/ksa.12785 (PMC12684322; doi:10.1002/ksa.12785)
Supplement: Supplementary file 1 — Appendix S1. [file KSA-33-4324-s001.docx]

**Appendix 1**

***Table 1:*** *Diagnostic codes defining complications.*

| **Categories with ICD-10 codes** | **Complication** |
| --- | --- |
| ***Medical complications*** | |
| Myocardial infarction | |
| I21n | Acute myocardial infarction |
| Cerebrovascular accident | |
| I63n | Cerebral infarction |
| Acute respiratory failure | |
| J952 J958  J96n R06 R060n R061 R064 R068 R092 | Acute pulmonary insufficiency following nonthoracic surgery Other intraoperative and postprocedural complications and disorders of respiratory system, not elsewhere classified  Respiratory failure Abnormalities of breathing Dyspnea Stridor Hyperventilation Other abnormalities of breathing Respiratory arrest |
| Pulmonary edema/Heart failure | |
| E877 J81 J819 I50 I501n I509 | Fluid overload Pulmonary edema Pulmonary edema, not specified Heart failure Left ventricular failure Heart failure, unspecified |
| Pneumonia | |
| J13n  J14n  J15n  J16n  J18, J180, J181, J188, J189  J690  J954 | Pneumonia due to Streptococcus pneumoniae Pneumonia due to Hemophilus influenzae Bacterial pneumonia, not elsewhere classified Pneumonia due to other infectious organisms, not elsewhere classified  Pneumonia, unspecified organism  Pneumonitis due to inhalation of food and vomit Chemical pneumonitis due to anaesthesia |
| Sepsis | |
| A021  A227 A267 A327 A40n A41n A427 A548G (6) A499 802n B377 | Salmonella sepsis Anthrax sepsis Erysipelothrix sepsis Listerial sepsis Streptococcal sepsis Other sepsis Actinomycotic sepsis Gonococcal sepsis Bacterial infection, unspecified Infections following infusion, transfusion and therapeutic injection Candidiasis-sepsis |
| Urinary tract Infection | |
| A560C A560D N109n N12 N129 N136n N151n N159 N16 N288n N30 N300 N309 N340n N341, N342, N390n | Chlamydial cystitis and urethritis Acute tubulo-interstitial nephritis Tubulo-interstitial nephritis, not specified as acute or chronic Pyelonefritis, Pyonephrosis Renal and perinephric abscess Renal tubulo-interstitial disease, unspecified Renal tubulo-interstitial disorders in diseases classified elsewhere Other specified disorders of kidney and ureter Cystitis Acute cystitis Cystitis, unspecifiedUretralabsces Urethral abscess Nonspecific urethritis Other urethritis Urinary tract infection, site not specified |
| Pulmonary embolism/Deep vein thrombosis | |
| T800 I80 I801n I802n I803n I808n I809 I82 I822n I828 I829n I26n | Air embolism following infusion, transfusion and therapeutic injection Phlebitis and thrombophlebitis Phlebitis and thrombophlebitis of femoral vein Phlebitis and thrombophlebitis of other and unspecified deep vessels of lower extremities Phlebitis and thrombophlebitis of lower extremities, unspecified Phlebitis and thrombophlebitis of other sites Phlebitis and thrombophlebitis of unspecified site Other venous embolism and thrombosis Embolism and thrombosis of vena cava and other thoracic veins Embolism and thrombosis of other specified veins Embolism and thrombosis of unspecified vein Pulmonary embolism |
| **Surgical complications** | |
| Mechanical | |
| T840n  T843  T844  T813 (C/D/S)  S830 | Mechanical complication of internal joint prosthesis  Mechanical complication of other bone devices, implants and grafts  Mechanical complication of other internal orthopedic devices, implants and grafts  Disruption of wound, not elsewhere classified  Subluxation and dislocation of patella |
| Infection | |
| T845n  T847  T814n | Infection and inflammatory reaction due to internal joint prosthesis  Infection and inflammatory reaction due to other internal orthopedic prosthetic devices, implants and grafts  Infection following a procedure |
| Fractures | |
| S723  S821n  M966 | Fracture of shaft of femur  Fracture of upper end of tibia  Fracture of bone following insertion of orthopedic implant, joint prosthesis, or bone plate |
| Unspecific | |
| T848  T849 | Other specified complications of internal orthopedic prosthetic devices, implants and grafts  Unspecified complication of internal orthopedic prosthetic device, implant and graft |

ICD; international Classification of Disease Version 10
*(“n” signifies all other possible sublevel values).
Inspired by* [1]

*Table 2: Procedure codes for identification of reoperations within 2 years from index surgery*

| **Categories with ICD-10 codes** | **Reoperation** |
| --- | --- |
| ***Stiffness/adhesions*** | |
| \| KNGH30 \| \| --- \| \| KNGH31 \| \| KNGH32 \| \| KNGT19 \| | Closed release of adhesions in the knee  Arthroscopic release of adhesions in the knee  Open release of adhesions in the knee  Manipulation under anaesthesia |
| ***Infection with DAIR*** | |
| \| KNGW59 \| \| --- \| \| KNGW69 \| | Reoperation for superficial infection after surgery on the knee or lower leg  Reoperation for deep infection after surgery on the knee or lower leg |
| ***Medial unicompartmental knee arthroplasty*** | |
| \| KNGB0  KNGB01 \| \| --- \| \| KNGB1 \| \| KNGC0 \| \| KNGC1  KNGC02  KNGC04 \| \| KNGC09  KNGB01 \| \| KNGC19 \| | Primary insertion of uncemented partial prosthesis in the knee joint Primary insertion of medial uncemented partial prosthesis in the knee joint Primary insertion of cemented partial prosthesis in the knee joint Secondary insertion of uncemented partial prosthesis in the knee joint Secondary insertion of cemented partial prosthesis in the knee joint  Secondary insertion of distal component in uncemented partial prosthesis in the knee joint Secondary insertion of more than one component in uncemented partial prosthesis in the knee joint  Secondary insertion of uncemented partial prosthesis in the knee joint, no specification Primary insertion of medial uncemented partial prosthesis in the knee joint  Secondary insertion of cemented partial prosthesis in the knee joint, not specified |
| ***Bleeding*** | |
| \| KNGW79 \| \| --- \| \| KNGW89 \| | Reoperation for superficial bleeding after surgery on the knee or lower leg  Reoperation for deep bleeding after surgery on the knee or lower leg |
| ***Wound rupture*** | |
| KNGW49 | Reoperation for wound rupture after surgery on the knee or lower leg |
| ***Unspecific*** | |
| \| KNGW \| \| --- \| \| KNGW99 \| | Reoperations after surgery on the knee and lower leg  Other reoperation after surgery on the knee or lower leg |
| ***Fracture*** | |
| KNGJ1  KNGJ2  KNGJ3  KNGJ4  KNGJ5  KNGJ6  KNGJ7  KNGJ8  KNGJ9 | Open reduction of fracture in the knee or lower leg  External fixation of fracture in the knee or lower leg  Internal fixation of fracture in the knee or lower leg  Internal fixation of fracture in the knee or lower leg with wires, rods, cerclage, or pins  Internal fixation of fracture in the knee or lower leg with intramedullary nail  Internal fixation of fracture in the knee or lower leg with plate and screws  Internal fixation of fracture in the knee or lower leg with screws alone  Internal fixation of fracture in the knee or lower leg with another or combined method  Other surgical fracture treatment in the knee or lower leg |

1. Jensen CB, Troelsen A, Foss NB, Nielsen CS, Lindberg-Larsen M, Gromov K (2023) No difference in short-term readmissions following day-case vs. one overnight stay in patients having hip and knee arthroplasty: a nationwide register study of 51,042 procedures from 2010-2020. Acta Orthop 94:516–522
