# Supplementary material for: Optimising outcomes in lateral unicompartmental knee arthroplasty: Analysing 25 years of registry data
Source: Knee Surg Sports Traumatol Arthrosc. 2025 Jul 13;33(12):4324–34. doi: 10.1002/ksa.12785 (PMC12684322; doi:10.1002/ksa.12785)
Supplement: Supplementary file 2 — Appendix S2. [file KSA-33-4324-s002.docx]

**Appendix 2**

***Table 1:*** *Cumulative risk of revision comparing TKA and lateral UKA (1997-2022)*

|  | **TKA** | | **Lateral UKA** | |
| --- | --- | --- | --- | --- |
| **Time** | **Risk estimate (95%CI)** | **%** | **Risk estimate (95%CI)** | **%** |
| 2 | 0.031 (0.024-0.039) | 3.1 | 0.073 (0.049-0.097) | 7.3 |
| 5 | 0.050 (0.036-0.056) | 5.0 | 0.101 (0.071-0.130) | 10.1 |
| 7 | 0.051 (0.040-0.062) | 5.1 | 0.106 (0.075-0.136) | 10.5 |
| 10 | 0.059 (0.046-0.072) | 5.9 | 0.136 (0.094-0.178) | 13.6 |
| 15 | 0.059 (0.046-0.072) | 5.9 | 0.175 (0.116-0.234) | 17.5 |

*Time, in years from index surgery. TKA: Total knee arthroplasty, UKA: Unicompartmental knee arthroplasty.
Death is incorporated as a competing risk for revision.*

***Table 2:*** *Absolute cumulative risk in % (95%CI) of revision for TKA and lateral UKA during different periods*

| **1997-2006** | | | **2007-2011** | | |
| --- | --- | --- | --- | --- | --- |
|  | **TKA (n=24)** | **UKA (n=8)** |  | **TKA (n=294)** | **UKA (n=74)** |
| 5y | 4.6 (0.0-13.2) | 25.0 (0.0-55.0) | 5y | 7.6 (4.5-10.7) | 16.2 (7.8-24.7) |
| 10y | 4.6 (0.0-13.2) | 50.0 (15.4-84.7) | 10y | 8.7 (5.4-12.0) | 18.9 (10.0-27.9) |
| *p=0.001* | | | *p=0.001* | | |
| **2012-2016** | | | **2017-2022** | | |
|  | **TKA (n=425)** | **UKA (n=107)** |  | **TKA (n=1379)** | **UKA (n=349)** |
| 5y | 4.7 (2.7-6.7) | 10.3 (4.5-16.1) | 5y | 3.7 (2.6-4.9) | 7.3 (3.9-10.6) |
| 10y | 6.4 (3.9-8.9) | 12.0 (5.5-18.6) | 10y | ## | ## |
| p=0.043 | | | p=0.011 | | |

*5- and 10-year cumulative risks of revision during different periods.
TKA: Total knee arthroplasty, UKA: Unicompartmental knee arthroplasty.*

***Table 3:*** *Distribution of 90-day complications for TKA and lateral UKA*

| **90-day complications** | | |
| --- | --- | --- |
|  | TKA n =2152, n (%) | Lateral UKA n = 538, n (%) |
| *Medical* |  |  |
| Myocardial infarction | 5 (0.2) | 1 (0.2) |
| Cerebrovascular accident | 7 (0.3) | 1 (0.2) |
| Acute respiratory failure | 3 (0.1) | 0 (0.0) |
| Pulmonary oedema/heart failure | 16 (0.7) | 6 (1.1) |
| Pneumonia | 12 (0.6) | 3 (0.6) |
| Sepsis | 5 (0.2) | 1 (0.2) |
| Urinary tract infection | 16 (0.7) | 5 (0.9) |
| Pulmonary embolism/deep vein thrombosis | 24 (1.1) | 9 (1.7) |
| *Surgical* |  |  |
| Mechanical | 67 (3.1) | 11 (2.0) |
| Infection | 39 (1.8) | 11 (2.0) |
| Fracture | 8 (0.4) | 0 (0.0) |
| Unspecific | 6 (0.3) | 5 (0.9) |

*TKA: Total knee arthroplasty, UKA: Unicompartmental knee arthroplasty.*

***Table 4:*** *Reasons for reoperations within the first two years after surgery for TKA and lateral UKA (1997-2022)*

| **Reason for reoperation, n (%)** | **TKA, n=2152** | **LAT UKA, n=538** |
| --- | --- | --- |
| MUA | 88 (4.1) | 3 (0.6) |
| Infection with DAIR | 10 (0.5) | 2 (0.4) |
| Medial UKA | 0 (0.0) | 0 (0.0) |
| Bleeding | 1 (0.1) | 0 (0.0) |
| Wound rupture | 0 (0.0) | 0 (0.0) |
| Unspecific | 1 (0.1) | 2 (0.4) |
| Fracture | 0 (0.0) | 0 (0.0) |

*TKA: Total knee arthroplasty, LAT UKA: Lateral unicompartmental knee arthroplasty, MUA: Manipulation under anaesthesia.
One knee can have experienced more than one reoperation and thereby be present more than once in the table.*
